# US food allergy patients’ experiences, priorities, and needs: A qualitative study

**DOI:** 10.1016/j.jacig.2025.100482

**Published:** 2025-04-22

**Authors:** Elisabeth Oehrlein, Silke C. Schoch, Omar A. Escontrías, Melanie Carver, Sanaz Eftekhari, Jessica Marvel, Chris Leo Pashos, Michael Pistiner, Randall Rutta, Jill Voss, Joe Vandigo

**Affiliations:** a Applied Patient Experience, LLC, Washington, DC; b National Health Council, Washington, DC; c Asthma and Allergy Foundation of America, Arlington, Va; d Novartis Pharmaceuticals, East Hanover, NJ; e Independent Advisor, Boston, Mass; f Mass General for Children, Boston, Mass; g Novartis Pharma AG, Basel, Switzerland

**Keywords:** IgE-mediated FAs, patient experiences, diagnosis pathways, health system experiences, psychosocial impact, personalized care

## Abstract

**Background:**

Understanding patient experiences with food allergies (FAs) is essential for developing treatments that meet patients’ needs and aiding clinicians in optimizing care.

**Objective:**

We sought to provide clinicians and researchers with an inclusive view of IgE-mediated FA patients’ experiences, including (1) FA’s natural history, including signs, symptoms, health system experiences, and diagnosis pathways; (2) the disease’s burden and psychosocial impacts on individuals and families; (3) perspectives on treatment attributes, benefits, and clinical trial participation; and (4) topics and resources patients and caregivers seek regarding FAs. Enhanced understanding of these challenges and opportunities can improve personalized care and patient outcomes.

**Methods:**

We conducted virtual interviews with 81 US residents with at least 1 physician-confirmed IgE-mediated FA. Interviews lasted up to 60 minutes. All participants were required to self-report a history of severe allergic reactions. Recruitment was via social media, health care providers, and patient advocacy organizations.

**Results:**

Most participants had multiple FAs, with peanuts and/or tree nuts (86%) being the most common. Common reaction types were skin/mouth (91%), respiratory (65%), and gastrointestinal (53%). Key diagnosis pathways included seeking urgent/emergency care after severe reactions and discussing moderate symptoms with primary care doctors. Barriers to diagnosis included long allergist wait times, travel, and other commitments. Most participants with epinephrine autoinjectors felt confident using them due to prior training. FA impacts varied, from minor daily disruptions to significant emotional, social, educational, and career challenges, mainly due to allergen avoidance efforts. Most participants (59%) showed an interest in clinical trials, influenced by potential risks and benefits, trial type, location, accessibility, and child willingness. Despite valuing epinephrine autoinjectors, participants noted unmet needs such as inconvenience, high costs, short expiration dates, and shortages. Desired treatment benefits included proactive treatments enabling allergen ingestion without symptoms and more information on FA management.

**Conclusions:**

This study offers insights into the diverse needs of IgE-mediated FA patients, highlighting the importance of comprehensive clinician awareness to enhance personalized care, optimize patient experiences, and improve outcomes. Addressing financial barriers and unmet needs through a team approach, clear communication, psychosocial support, and accessible resources is essential.

A food allergy (FA) is “an adverse health effect arising from a specific immune response that occurs reproducibly on exposure to a given food.”^1^ Disagreement regarding diagnostic criteria and a lack of high-quality population-level data make identifying reliable prevalence estimates challenging.^2^ The Centers for Disease Control and Prevention estimates that the US prevalence is 6.2% (∼20 million people).^2^^,^^3^ A cross-sectional, population-based survey fielded in 2015-2016 estimated childhood FA prevalence at 7.6%.^4^ In addition to challenges documenting prevalence, there is a lack of comprehensive information on the natural history of FAs.^1^ FAs are classified as IgE-mediated, non–IgE-mediated, or mixed mechanisms. IgE-mediated allergic reactions typically occur shortly after exposure and can cause mild to potentially fatal food-induced anaphylaxis. Most IgE-mediated allergic reactions are caused by milk, egg, peanut, soy, wheat, tree nuts, shellfish, fish, and sesame seeds.^5^, ^6^, ^7^, ^8^ They are diagnosed on the basis of a history of classic clinical symptoms and diagnostic testing by either skin prick or serum-specific IgE blood testing.^9^ Because skin prick and blood tests are prone to false positives, oral food challenges (OFCs) are considered the criterion standard in determining whether a person can safely eat a food.^10^ However, OFCs are time and resource-intensive, carry a risk for severe allergic reactions, and are associated with “anxiety among patients, families, and even health care professionals.”^11^^,^^12^

There is no cure for FA. Treatment includes elimination diets, epinephrine for severe reactions, and antihistamines to manage nonsevere symptoms.^5^ To date, 2 FA treatments are approved by the US Food and Drug Administration (FDA). Peanut (*Arachis hypogaea*) Allergen Powder-dnfp, an oral immunotherapy (OIT), is indicated for a small subset of children with peanut allergies, and the FDA requires health care providers (HCPs) to have Risk Evaluation and Mitigation Strategies certification to prescribe it.^13^^,^^14^ Omalizumab received an expanded indication to reduce severe FA reactions among people with IgE-mediated allergies. It is intended to be used in conjunction with food allergen avoidance.^15^ Other forms of OIT have been tested in research settings. However, adoption in clinical practice is limited by the number of HCPs offering it, safety concerns, and inconvenience.^11^^,^^16^ Several therapies are under development to reduce or prevent symptoms of allergic reactions.^17^

The FDA recognizes that people living with a chronic condition/disease and their family caregivers are “uniquely positioned to inform the understanding of the therapeutic context for drug development and evaluation.”^18^ Comprehensively understanding patient experiences living with and treating FAs is key to developing medical products and treatments that fill an unmet need as defined by patients. These data can also help clinicians better understand their patients to optimize care. In a recent *Journal of Allergy and Clinical Immunology* article, researchers emphasize the importance of including diverse FA patient populations in advocacy, research, and education.^19^

Thus, the purpose of this study was to offer clinicians an inclusive look at IgE-mediated FA patient experiences, including (1) the natural history of FA, including signs and symptoms, health system experiences, and pathways to diagnosis; (2) the burden of disease and psychosocial impact of FAs on individuals and families; (3) perspectives on meaningful treatment attributes and benefits, and clinical trial participation; and (4) topics patients and caregivers would like to learn more about and resources that they use to navigate FAs. By better understanding patient challenges and opportunities, clinicians can enhance personalized care to improve patient outcomes.

## Methods

Patient experience data, consistent with FDA expectations, were collected via interviews among adults, adolescents, and adult caregivers of children with physician-confirmed IgE-mediated FA.^20^ Interviews collected holistic patient experience data about (1) FA natural history; (2) personal experiences related to diagnosis and treatment; (3) burden on patients and families; (4) perspectives on meaningful treatment benefits; (5) sources of information; and (6) perspectives on clinical research participation. This study was guided by a multidisciplinary Steering Committee, which included patient advocates and a physician specializing in FA.

### Participants

Our target patient population included people with at least 1 IgE-mediated FA (milk, egg, peanut, soy, wheat, tree nut, shellfish, fish, and sesame) and self-reported history of severe allergic reaction. All participants were required to meet the eligibility criteria listed in Table I. Participants were recruited via social media, referrals from HCPs, and patient advocacy organizations. Eligibility was validated by evidence of a physician-confirmed diagnosis through a physician’s note, health records, school note, or photos of their epinephrine autoinjector.

**Table I:** Eligibility criteria

**Inclusion criteria**
1. Individual with a confirmed diagnosis or a family caregiver for a child with a confirmed diagnosis • Adults (ADU): at least 18 y of age or the age of majority in the state they reside in • Adolescents (ADOL): 12-17 y of age • Family caregivers (CG) of young children younger than 12 y of age 2. One of the following: • History of a severe reaction requiring urgent care or emergency room visit, or hospitalization related to an FA; or • Severe reaction that required the use of epinephrine; or • Severe reaction that in retrospect should have resulted in a visit to urgent care clinic or emergency room 3. FA impacts their daily routine, such as school, work or social life, or mental health 4. FA was diagnosed at least 6 mo ago 5. Able to participate in an interview lasting ∼60 min 6. Able to read and communicate in US English 7. Resides in the United States 8. Access to an internet connection with a laptop, desktop, cell phone, or tablet
**Exclusion criteria**
1. Blindness or significant visual impairment 2. Participants who self-report comorbid food protein–induced enterocolitis syndrome and/or inflammatory bowel disease and/or diabetes type I or II 3. Currently incarcerated 4. Patients who did not meet the inclusion criteria

### Ethics

This study was approved by Advarra’s Institutional Review Board (Pro00067063). Participants were compensated $135 for their participation, which aligns with the National Health Council’s Patient Engagement Fair-Market Value Calculator.^21^

### Study procedures

We adapted the National Health Council Patient Experience Mapping Toolbox interview guide to include questions about FA patient experiences.^22^ The Patient Experience Mapping Toolbox was developed to help researchers capture patient experience data across chronic diseases. The interview guide, consent forms, and other patient-facing materials were developed by patients, patient advocates, and other experts, reviewed externally by health literacy experts, and refined through patient interviews.

All interviews were conducted virtually and lasted up to 60 minutes. Participants could decide to be on or off-camera. Interviews were conducted between November 2022 and March 2023 (note omalizumab was not yet FDA-approved).

Interviews were audio-recorded, transcribed verbatim, and coded line-by-line using a hybrid deductive and inductive approach. Deductive codes were based on stages in the “Map My Experience” conceptual model (before diagnosis, getting a diagnosis, and living with a confirmed food allergy) and prespecified questions, such as which diagnostic tests are used at diagnosis. Inductive codes emerged on the basis of participant answers to open-ended questions. We used a team-based approach to coding qualitative data (led by coauthor E.M.O.).^23^ Coding was conducted in MaxQDA (Qualitative Data Analysis Software [Berlin, Germany]).

Participant characteristics were summarized using descriptive statistics. Continuous variables were reported as means with SDs or medians with interquartile ranges. Categorical variables were presented as frequencies and percentages. The methods and interim results were presented and discussed among the project team during biweekly meetings and quarterly with the Steering Committee.

## Results

We interviewed 81 people residing in the United States with at least 1 physician-confirmed diagnosed IgE-mediated FA. Participant characteristics are reported in Table II. More than half were diagnosed before the age of 7 years (Table II). Many participants had multiple FAs, and the most common allergies were to peanuts and/or tree nuts (86%), with allergies to milk and/or shellfish each found in 27% of respondents. Younger participants were more likely to report a peanut allergy, and older participants were more likely to report a shellfish allergy. Self-reported reaction types were largely skin or mouth (91%), respiratory (65%), and gastrointestinal (53%).

**Table II:** Participant demographic and clinical characteristics

Characteristic	Total (N = 81)	Caregivers∗ (N = 27)	Adolescents (N = 27)	Adults (N = 27)	*P* value
Age today (y), mean ± SD	21.6 ± 17.4	7.4 ± 3.1	14.6 ± 1.8	42.9 ± 13.8	<.001
Age at diagnosis (y), mean ± SD	9.6 ± 12.4	2.6 ± 2.9	4.7 ± 4.0	21.5 ± 15.1	<.001
Sex					.37
Male	32 (40)	13 (48)	11 (41)	8 (30)	
Female	49 (60)	14 (52)	16 (59)	19 (70)	
Race					.22
Asian	9 (11)	3 (11)	5 (19)	1 (4)	
Black or African American	18 (22)	4 (15)	5 (19)	9 (33)	
Other	5 (6)	0 (0)	3 (11)	2 (7)	
Two or more	3 (4)	2 (7)	1 (4)	0 (0)	
White	46 (57)	18 (67)	13 (48)	15 (56)	
Hispanic or Latino					.86
No	74 (91)	25 (93)	24 (89)	25 (93)	
Yes	7 (9)	2 (7)	3 (11)	2 (7)	
Community setting					.74
Rural	8 (10)	3 (12)	2 (7)	3 (11)	
Suburban	43 (54)	14 (54)	17 (63)	12 (44)	
Urban	29 (36)	9 (35)	8 (30)	12 (44)	
Census region					.94
Midwest	22 (27)	7 (26)	9 (33)	6 (22)	
Northeast	20 (25)	7 (26)	7 (26)	6 (22)	
South	24 (30)	8 (30)	6 (22)	10 (37)	
West	15 (19)	5 (19)	5 (19)	5 (19)	
Household income					.36
<$25,000	3 (4)	1 (4)	0 (0)	2 (7)	
$25,000-$49,000	14 (18)	5 (19)	5 (19)	4 (15)	
$50,000-$99,000	21 (26)	8 (31)	6 (22)	7 (26)	
$100,000-$149,000	22 (28)	5 (19)	6 (22)	11 (41)	
≥$150,000	20 (25)	7 (27)	10 (37)	3 (11)	
Allergy type					
Peanut	37 (46)	13 (48)	17 (63)	7 (26)	.023
Tree nut	32 (40)	11 (41)	14 (52)	7 (26)	.15
Milk	22 (27)	9 (33)	10 (37)	3 (11)	.068
Shellfish	22 (27)	3 (11)	6 (22)	13 (48)	.007
Fish	12 (15)	3 (11)	4 (15)	5 (19)	.75
Wheat	9 (11)	3 (11)	2 (7)	4 (15)	.69
Egg	8 (10)	3 (11)	4 (15)	1 (4)	.38
Soy	5 (6)	2 (7)	2 (7)	1 (4)	.81
Sesame seeds	4 (5)	3 (11)	1 (4)	0 (0)	.16
Reaction type					
Skin and/or mouth	74 (91)	26 (96)	27 (100)	21 (78)	.008
Respiratory	53 (65)	14 (52)	17 (63)	22 (81)	.069
Gastrointestinal	43 (53)	17 (63)	19 (70)	7 (26)	.002
Cardiovascular	4 (5)	2 (7)	0 (0)	2 (7)	.35
Neurological	2 (2)	1 (41)	0 (0)	1 (41)	.60
Comorbidities					
Asthma	21 (26)	8 (30)	6 (22)	7 (26)	.82
Eczema	17 (21)	9 (33)	5 (19)	3 (11)	.12
Hay fever or allergic rhinitis	17 (21)	2 (7)	7 (26)	8 (30)	.099
Proctocolitis	0 (0)	0 (0)	0 (0)	0 (0)	
Eosinophilic esophagitis	1 (1)	0 (0)	0 (0)	1 (4)	.36
Oral allergy syndrome	4 (5)	1 (41)	2 (7)	1 (4)	.77
Mast cell activation syndrome	0 (0)	0 (0)	0 (0)	0 (0)	
Other allergies	13 (16)	5 (19)	4 (15)	4 (15)	.91
Other comorbidity	6 (7)	3 (11)	1 (4)	2 (7)	.58

Values are n (%) unless otherwise indicated.

∗ Caregivers reported demographics on behalf of their child.

### Natural history of FA

About 10% of interviewees had trouble remembering the specifics of their diagnosis because it occurred at a very young age, sometimes decades ago. The most common starting point for peoples’ experiences with FAs was that someone outside the health system noticed signs or symptoms (see Table III). This was either the individual themselves, a family member, or a school or daycare provider. Parents mentioned that it can be challenging to identify FAs among infants and toddlers who cannot yet speak, which is significant given that 43% of diagnoses occurred before the age of 4 years. People experience a range of symptoms when they encounter a food allergen. Reactions range from mild symptoms to severe anaphylaxis requiring emergency care. Participants described symptoms characteristic of IgE-mediated (Table IV) and non–IgE-mediated FAs (see Online Repository Materials, Table E1, at www.jaci-global.org). Direct quotes from patients provide examples of the terminology patients use to describe symptoms.

**Table III:** Common starting point for patient journey with FAs

Starting point	Illustrative quotes from interviewees
Individual notices symptoms	**Adult** (shellfish): I noticed something was wrong because I was itching and breaking out in hives. **Adult** (peanut): Oh, it was definitely me. That’s for sure…. I had like an allergic reaction to peanuts. So, I ended up going to the hospital for that. Well, I had a really severe reaction. My throat swelled up, I had to call 911 and I had to go to the emergency room ASAP. **Caregiver of a young child** (tree nut): We were at home, he ate a quarter of a cashew and immediately vomited. He said his mouth hurt and he vomited immediately. He was just four years old, our daughter had been sick for a week so I thought maybe he’s just getting sick, it didn’t cross my mind it was a food allergy and then later in the night he had trouble breathing, so we went to the doctor the next day and as talking through with the doctor he suggested it could be a food allergy and did a blood test there.
Family member notices signs	**Caregiver of a young child** (peanut, tree nut): He was nine months old, my husband decided to feed him peanut butter, and he broke out in hives all over his body. **Adolescent** (tree nut, fish): My mom found the problem. I had really bad eczema as a baby so my mom got me tested.
School or daycare notices signs	**Caregiver of a young child** (peanut): She ate peanut butter pretty regularly and I ate it all the time… But she was at day care, and they served her a peanut butter sandwich … and her lip swelled. So, they called me and said “Hey, we wanted to let you know she’s eating a peanut butter sandwich, her lip started swelling so we took it away.” They gave her Benadryl at my request and then I went and picked her up and took her to our primary care which is also an urgent care through her children’s hospital, and they looked at her and sent us to get a blood test and that’s where the diagnosis came in.
Milk allergy discovered while breast- feeding or receiving infant formula	**Caregiver of a young child** (milk, peanut, tree nut, shellfish): My son was six weeks and he had breast milk jaundice, so what that means is that he’s a very healthy, thriving baby but he still had jaundice, and so the pediatrician told me to use formula, to flush out that. Then so I did, and it was really hard to feed him formula because he’s mostly breast-fed, and then he broke out in a rash in his face. I told the pediatrician that and then she said he may have a dairy allergy, to stop doing dairy.

**Table IV:** Signs and symptoms of IgE-mediated FAs

Symptom	Illustrative quotes from interviewees	Frequency, n (%)
Rash or hives	**Caregiver of a young child** (peanut, tree nut): [Child] actually broke out in a big case of hives. **Adult** (shellfish): I began to get hives and stuff on the top part of my arms, and then also on the lower part of my back.	50 (61.7)
Swelling	**Adult** (peanut): I had a really severe reaction. My throat swelled up, I had to call 911 and I had to go to the emergency room ASAP.	44 (54.3)
Choking, difficulty breathing, coughing	**Adult** (peanut): I was really upset at… I thought I was choking, but I didn’t understand why I was choking. I had no idea what was happening, to be honest with you, so that was really quite frightening. **Adolescent (peanut):** I remember being in the car and just feeling like I couldn’t breathe and really scared.	42 (51.9)
Itchiness	**Adult** (tree nuts, shellfish): My lips started itching, my tongue started itching, my throat started itching. **Adolescent** (peanut, shellfish): Any time that I’ve eaten peanuts, the top of my mouth starts to kind of swell up and get super itchy.	29 (35.8)
Gastrointestinal issues	**Adult** (milk): Vomiting, stomach cramps, dizziness, being clammy. **Caregiver of an adolescent** (peanut, tree nut, shellfish, fish): He loved it but every time after he ate it, he would go straight to the bathroom and be in there throwing up for like, 20, 30 minutes.	25 (30.9)
Eye-related symptoms	**Caregiver of a young child** (peanut): She keeps itching her eyes like something is wrong with her eyes. Her eyes were red and swollen. **Caregiver of a young child** (peanut, tree nut): He woke up and one of his eyes was swollen, completely shut, like he could not … it was completely shut swollen, I’d never seen anything like that.	6 (7.4)
Loss of consciousness	**Adult** (milk, peanut): It was quite critical, so I actually passed out, like fainted, because of the body, which was reacting. **Adult** (wheat, tree nuts): I start to get hot and then I black out. One time I was like oh, all I need to do is go and sit down in my car. I walked out of the event I was at. Unfortunately, I was at event so everybody got to watch all of these happen. Walked out of the event, stepped off the curb and fell faceplanted forward because I passed out while I was stepping off the curb. I was like, I know I’m not ready to drive home, but I just need to go sit down and I need to leave this event and just manage myself or get away from the craziness that’s going on around me.	4 (4.9)
Sleepiness or fatigue	**Caregiver of an adolescent** (peanut, tree nut, shellfish, fish): He starts to… he gets sleepy. He gets really tired after that and it concerns me that something’s going to happen to him when he’s asleep.	3 (3.7)

### Diagnosis experiences

Among our sample, the 2 most common pathways to diagnosis were (1) seeking urgent care or visiting an emergency room after experiencing a severe allergic reaction (37%) and (2) observing more moderate signs and symptoms over time and discussing them with a primary care doctor or pediatrician (43%).

Participants described several barriers to seeking or confirming a diagnosis, including long wait times to see an allergist, even if they had been to an emergency room for a severe allergic reaction, lengthy travel times, and interference with other commitments. Two participants described symptoms emerging during childhood but not receiving a formal diagnosis until adulthood due to insurance barriers.

Most interviewees explained that whether they sought immediate or primary care, they were ultimately referred to an allergist for testing or confirmation of diagnosis (83%), which included skin prick test only (29%), blood test only (16%), or both skin prick and blood tests (50%). Three participants (4%) who were unsure about their diagnostic testing journey stated that they received a diagnosis while still in the hospital following an anaphylactic reaction. Although not all participants were aware of the specific names of the blood tests they received, these people reported having a blood test under the supervision of an allergist, another physician, or in the hospital, followed by a discussion about their specific allergies 1 or 2 weeks later. Participants described being surprised to learn they had multiple allergies through diagnostic tests, leading some to question whether they were truly allergic or whether the results were false positives (19%). This uncertainty can give hope that they or their child might eventually “grow out” of certain allergies. However, it can also cause them to unnecessarily avoid nutritious foods they may not actually be allergic to.

Although few described undergoing an OFC (15%), when asked, it was apparent that there was confusion about the difference between an OFC and OIT.

### Treatment experiences

Nearly all participants received a prescription for an epinephrine autoinjector (90%) at some point, usually immediately upon diagnosis. However, not all participants picked up their prescriptions for reasons including shortages and out-of-pocket costs. For example, one adolescent participant described completing the IgE blood test but then losing health insurance. Others did not currently have epinephrine autoinjector prescriptions because they were not required to by their school, their “airways had never closed,” or they felt taking corticosteroids or antihistamines and avoiding allergens was sufficient. Those who had never received an epinephrine autoinjector were unsure why not or described various reasons, including rarely encountering the allergen or believing the reaction was not severe enough (eg, “He has never had that kind of a reaction where it has affected his breathing or anything, so they never gave him an Epi-Pen.”)

Among participants with an epinephrine autoinjector, most (70%) felt comfortable they would be able to use it in an emergency because they had previously used it, or their allergist had trained them. Parents stated that insurance typically covers a maximum of 2 at any one time—making it impossible for both parents, the child, and the school to have epinephrine autoinjectors simultaneously. In addition to epinephrine autoinjectors, many participants reported using antihistamines as their primary treatment for reactions or using antihistamines in tandem with epinephrine autoinjectors if an allergic reaction became severe (72%). In our sample, 14% of participants had tried OIT under the supervision of their allergist, whereas 6% of participants learned about OIT and tried an unsupervised version.

### Burden of FAs on individuals and families

Our sample comprised people with a self-reported history of severe allergic reactions. Although some interviewees felt that their FA only had a minor impact on their daily lives, others described significant impacts on their emotional health, social life, education, and career. In most cases, these impacts are largely the result of attempts to avoid the allergen.

Most participants (93%) stated that they avoided direct contact or consuming allergens (see Table V). Interviewed parents were particularly worried about their children consuming an allergen outside of the home. Although some parents try to prepare their children to speak up and avoid allergens, others go to great lengths to ensure their children do not encounter foods they are allergic to, including declining invitations to family gatherings and social events.

**Table V:** Approaches to daily activities and diet

Approach	Illustrative quotes from interviewees
*Daily activities*	
Avoid activities because of the potential of coming into contact with an allergen	**Caregiver for an adolescent** (milk): I guess I’m a little bit more protective of [participant’s name] like if he wants to join the basketball team, but it means he has to stay after school until 6 o’clock, then I’m like oh maybe wait until he’s a little bit older. So maybe I’m a little more protective, and I’ve kind of held back, I don’t want to push him to join the basketball team because I know it will be a challenge with the food allergies.
Careful, but participated in most activities outside of their home	**Adult** (shellfish): I just be careful when we go out to eat. Not to go to certain restaurants or not to eat certain food, and if I’m not sure what’s in it, then I just don’t eat it. **Caregiver of a young child** (sesame): We pack [patient’s name] lunch, we do not utilize the school food option, because we tried to embark in this two years ago, and it was just very difficult to get the listing of the ingredients for every food that would be coming up. So, we just in the end decided that we knew there were a couple of safe foods for her that if she wanted that hot school lunch option, she knows when those days come around, she can have it. But other than that, we package our own.
Participate in all activities even when it means encountering allergen	**Adult** (milk, peanut): So sometimes, for example, if we want to cook a meal for the whole family when there is a get together, for example, they see me like I make them they work harder because they have to make a different meal for me. So sometimes I overlook my effect or the reaction. I tell them just to make everything the same. I’ll take the medication after. So, I overlook my allergies. So, living with these kind of allergies sometimes it is quite hard. Especially when you are the only person who has it in a family.
*Dietary approach*	
Vigilant about ingredients and food labels	**Adolescent** (peanut): I watch certain food when I’m around people during lunch. And if they try to, want to offer me food I have to say no, or is that peanuts? So, I just have to always be aware of what the food has it in. **Caregiver to an adolescent** (peanut, tree nut): When she got of the age that she could read, no matter if there was something brought in, she would read the ingredients herself and make sure that they don’t in fact have nuts. When she was younger and she could not read, even for potato chips or even hard candy she would ask, does this have nuts, does this have nuts? And that’s how we told her in order for her to be safe when her parents are not around she’d have to continue to do that.
Less careful and choose to eat foods with cautionary labels	**Adolescent** (tree nut): I’ve eaten things and I’m like, I’m just going to like [laughs]… you eat a candy bar that says processed in the same factory as almonds, that kind of risk it to see what happens. Sometimes you lose your voice and sometimes nothing happens at all. I’m probably frowned upon to do that.
Choose to eat foods they are allergic to	**Adolescent** (peanut, soy): I still eat bread and stuff willingly. So, probably once or twice a week… Yeah. It’s bad, Sometimes, I’ll get a rash. So, I’ve been trying to not eat bread as much now. I usually use hydrocortisone. I’ve got hydrocortisone or my mom, she has this ointment that she got for rashes and stuff so I’ll use that.

Less than half the participants (40%) described that FAs have little impact on their finances, attributing this to good insurance coverage of care and treatment. Others described how FAs and the broader health system are a financial burden (see Table VI). Participants stated that growing awareness about FAs has made finding substitutes for common allergens at the grocery store easier. However, substitutes are often more expensive and can be difficult to purchase in rural settings. In addition, substitutes may contain other common allergens, presenting an additional burden for those with multiple FAs. For example, an individual with wheat and tree nut allergies commented that wheat/gluten-free substitutes are available in most grocery stores, but unfortunately, many contain almond flour, a tree nut to which they also have an allergy.

**Table VI:** Illustrative examples of the financial burden of managing FAs

Financial impact	Illustrative examples from interviewees
High costs of food and special accommodations	**Adolescent (**egg, tree nut, sesame): It’s more expensive to always have to cook, and to get hotels rooms that have kitchens, so I think it’s costly. Auvi-Qs are expensive. And we have to continually renew the prescription, just to have them on hand. RX coupons help with affordability. **Caregiver of a young child** (milk, peanut, tree nut): I don’t even know what our grocery bill is up to now with inflation; however, I have worried, even my church has a food bank, I did the math recently and said this large percentage of kids have food allergies.
Special formula for babies with a milk allergy	**Caregiver of a young child** (milk, soy, wheat): It was a challenge financially with the formula she was on and the restrictions that we had in her diet. She pretty much is only supposed to eat raw fruits, vegetables, and meats… I did go through several jobs just because of the amount of time that [patient’s name] needed when she was younger to be in the hospital and then all of the appointments we had to make trying to get results and a diagnosis.
High costs of health care, especially without insurance	**Adult** (shellfish): Yeah, I didn’t have any health care insurance and I have bills now, I have health care bills that I have not paid and just can’t pay them right now, so yeah, my credit has been affected. Just that one hospital ride, they charged for the ambulance, they charged for you being in there, and every time after that.
Medication costs, especially of epinephrine autoinjectors, which need to be repurchased regularly and multiple epinephrine autoinjectors are not always covered by insurance	**Adult** (shellfish): I have a wonderful health insurance group, but I would say to private ones to stop being so stingy with EpiPens. I have friends that are begging me to have one of my EpiPens because theirs are hundreds of dollars even with their insurance. It’s ridiculous. **Caregiver of a young child** (tree nut): EpiPen started charging $500 a shot. We dealt with all that and then it was like, if any… I think they last, or they’re only proved effective for up to six months. So, every six months we need roughly four to five EpiPens, one for school, one for each of us, one for each car. So that, at the time, we went through two rounds where it was close to $2,500 just to get EpiPens.
Seeing specialists & diagnostic and laboratory tests	**Adult** (tree nut, shellfish): So every time I visited the allergist I had to take a breath test, so I had to pay a co-pay, plus the breath test. I think together it was 55, every time I saw her. The lab tests. I forgot at the time how much I paid for them but these weren’t like routine things that the allergist was doing for me, so I definitely paid for the skin test. I paid for the blood test, which I think it was 250, so financially I was spending a lot of money just focusing on my allergies and things, that year. 2020 and parts of 2021, yeah, 2019, and then 2020 I spent a lot of money.

### Future treatments and meaningful treatment benefits

Although participants were all grateful that epinephrine autoinjectors are available, they expressed unmet needs, including inconvenience, high out-of-pocket costs, short expiration dates requiring frequent replacement, and supply shortages. In terms of meaningful treatment benefits that are not a cure, the most common response was a desire for “proactive” treatments allowing them to ingest an allergen without developing symptoms or to be able to be around others who are eating the food.

“I would like a treatment that would allow me to eat any food that I wanted to, or taste new foods, or feel comfortable around friends that do have foods with peanuts or tree nuts.” - Adolescent with milk, peanut, and tree nut allergies

When asked about interest in participating in a clinical trial, more than half (59%) stated they were interested or potentially interested. Table VII describes participant experiences and perspectives on clinical trials for treatments related to FAs, including factors influencing interest in trial participation. These include the potential risks and benefits, the type of trial or treatment being offered, the location and accessibility of the trial, and willingness of the child to participate.

**Table VII:** Participant experiences and perspectives on clinical trials for treatments related to FAs

Experience or perspective	Illustrative quotes from interviewees
Currently participating in a clinical trial	**Exciting, but inconvenient:** • **Caregiver of a young child** (milk, peanut, tree nut): The only downside is we live 5-6½ hours away from the doctor in [location] and so he has to travel every 4 weeks or so and usually miss school to go and get the treatment and it’s about to increase, but it is allowing him, it has changed the threshold for his allergies so for the first time in his life he can actually be more like a little kid, without the terrifying reactions.
Familiar with FA trial and potentially interested, but barriers	**Inconvenient:** • **Caregiver of an adolescent** (peanut): I think it’s just been he’s been so busy. And I know with the other thing I was telling you about, it’s how it was a weekly commitment, that we have friends that have done it and we weren’t able to… it’s a 45-minute drive from where we are since there’s so few doctors that do it. I guess we would be open to it if it’s something that worked with his schedule. **Did not meet severity eligibility criteria:** • **Adult** (peanut, tree nut, shellfish, fish): The person who was interviewing me to qualify said my allergies were not severe enough, but my current allergist disagrees with that and said that I have multiple allergies that would’ve qualified. **Risks outweighed potential benefits of trial participation:** • **Adolescent** (peanut): I felt like the trial was a little too risky and I didn’t want to risk anything. Because what you have to do is they would give you a little bit of a peanut. And at first, I would take it at the hospital, but then I would have to start taking it at home. And I just felt like that was too much of a risk for me, so I decided that I wouldn’t want to do it. ○ **Caregiver for the same adolescent (**peanut): We didn’t want her to have more anxiety. And like she was saying, we were also afraid because it wasn’t only going to be when we did the food challenges, it wasn’t only going to be in a safe environment, it was going to be something we had to do at home. And then she gave us some of the statistics that she might be affected by it, she might have to use her EpiPen, they couldn’t guarantee us anything, she would need to do another food challenge before they could even start the trial. And at this point, we tried to really look at her and see what she felt like, because she’s old enough at this point. But she didn’t want to do it, and we really weren’t comfortable either.
Potential willingness to participate	**Willing to participate, but have not been asked:** • **Adult** (wheat, tree nut): I’d be open to it. I’m in clinical trials for my GI symptoms. So, I’ve done also clinical trials for [university name] and a few other, the [clinic name] and a few others. **Unfamiliar with clinical trials for FAs:** • **Caregiver for an adolescent** (milk, peanut): No, we haven’t heard anything… I know there’s clinical trials for cancer and stuff, but I’ve never heard of a clinical trial for peanuts. Now, is that where they would test her, or have her try something, or something?
Not interested	**FA largely under control:** • **Adult** (shellfish): Not really, no. I’ve gotten used to it by now. I don’t feel as if I’m missing out on much.

*GI*, Gastrointestinal.

### Questions and information sources

When asked where they look for information about FAs, participants mentioned the internet (eg, WebMD, Mayo Clinic, and TikTok), HCPs, family and friends, scientific literature, books, and support groups, including online forums and patient organizations.

Table VIII presents topics they would like to learn more about and resources that would be helpful to them. Participants wished they had known about their FAs when they first noticed signs and symptoms and expressed a desire for more information about warning signs and triggers, anaphylaxis management, and navigating daily life with an FA.

**Table VIII:** Topics and resources to develop or more widely distribute to people with FAs as suggested by interviewees

Topics	• Long-term effects of FAs • Connection between multiple FAs • Epinephrine autoinjector disposal • When and if it is okay to use someone else’s epinephrine autoinjector in an emergency • Navigating social situations to avoid allergens • Recipes and cooking tips • Introduction to immunotherapy • Information about clinical trials
Resource: Peer-to-peer support	**Caregiver of a young child** (peanut): It would have been nice to have, when he was diagnosed, it would have been really nice to have an appointment, counseling and education appointment with someone who was not his allergist but maybe like a support person who was very knowledgeable or maybe a parent who could understand what we going through and could tell us, “My child is now this old and this is what we do and this what works for us.”
Resource: Plain language resources available at point of care	**Adult** (shellfish): We need more information in layman’s terms… When I go to my allergist, I’ve never seen a pamphlet there that says, “Shellfish allergies. What to do for shellfish allergies.” I’ve never seen a booklet like that. I’ve never seen a booklet that says, “If you are allergic to dogs and cats, this is some of the stuff you need to do.” A booklet that can break it down from using certain laundry detergent to get rid of dander. Stuff like that, just simple book.
Resources for adults	**Adult** (peanut, tree nut, shellfish, fish): I also wish there were larger online support groups for people in my age demographic. Because there’s not a lot of resources for adults… it’s mostly for parents with children for allergies.

Finally, participants suggested that food and beverage companies and restaurants should be more conscientious toward people with FAs. For example, food and beverage companies must be more transparent about actual ingredients to support people with allergies. Unclear or nonspecific cautionary food labels may lead people with FAs to avoid foods unnecessarily.

## Discussion

This study provides insights into the experiences of people living with severe, physician-confirmed IgE-mediated FAs in the United States. Our findings describe humanistic aspects of the natural history of FAs, such as what guides patients’ decisions to seek care or how patients describe their own symptoms, individual and family impacts, and perspectives on future treatments. These findings contribute to a holistic understanding of the experiences and challenges faced by those with FAs. It can inform improvements to clinical practice and medical product development.

We found that the time between noticing signs and symptoms and entering the health system to seek a diagnosis is related to the severity of the first allergic reaction. Clinical practice guidelines stress that the severity of a reaction cannot be predicted solely on the basis of past reactions and risk factors.^5^ Relying only on oral antihistamines is associated with an increased risk of near-fatal or fatal reactions.^1^ Understanding care-seeking patterns, barriers, and the vernacular patients use to describe their symptoms may help primary care providers identify and recommend further testing among people with moderate reactions to date.

People with FA have varied life circumstances. A care team approach focusing on clear communication, psychosocial support, and awareness of existing patient-friendly resources may help address their unique unmet needs. Topics that clinicians and patients could discuss upon diagnosis include life circumstances, including the accessibility of allergy-safe foods, the ability to pay out-of-pocket costs, and whether patients have support in managing their FAs at home, school, and in the workplace.

Confusion and misconceptions about diagnostic tests suggest a need for clearer communication between HCPs and patients, as well as an opportunity to connect patients more efficiently with plain language resources that already exist. Examples included a belief that the higher numbers in diagnostic test results are correlated with more severe allergies. Past research indicates that patients may have an improved understanding of their care and are more motivated to adhere to care plans when clinicians provide printed summary information at the end of an appointment.^24^, ^25^, ^26^ Patient-friendly resources exist to address these misconceptions, as well as those topics participants suggested they would like to learn more about.^27^ For example, patient groups, including the Asthma and Allergy Foundation of America and Food Allergy Research & Education, provide FA education for patients and caregivers and maintain lists of allergy-friendly recipes.^28^, ^29^, ^30^, ^31^ Thus, there appears to be a disconnect between available resources and patients’ awareness of them.

Our study is consistent with research documenting the psychosocial burden of FAs among patients and their families.^32^, ^33^, ^34^ We found that the burden is largely due to lifestyle changes that many people with severe allergies make to reduce the risk of accidental exposure to an allergen. This echoes caregiver feedback and key takeaways collected during an externally led Patient-Focused Drug Development meeting, which highlighted parents’ persistent anxiety about their child experiencing an allergic reaction, feeling overwhelmed by fear of an anaphylactic reaction, and worries as children become more independent as they get older.^35^ Clinicians can help patients and their families by encouraging them, clearly explaining food allergies, the role of epinephrine, and as needed, consider referring patients and family members for emotional support.^36^^,^^37^ Participants also described the financial impacts of FA, consistent with a recently published economic burden study highlighting high out-of-pocket costs associated with having to purchase allergy-safe foods and medications.^38^

This study also confirmed that this therapeutic area has unmet medical needs as people with FAs and their caregivers seek convenient and efficacious treatments. For example, participants expressed frustration that epinephrine autoinjectors are “reactive.” Instead, participants sought “proactive” treatments that would reduce the severity of allergic reactions. This is comparable to the value of recent vaccines that have been shown to reduce the severity of various respiratory illnesses.^39^ This aligns with a recent Delphi panel of caregivers and people with FAs that identified reducing the risk of serious anaphylaxis or allergic reactions as a key high-value potential benefit of future treatments.^40^

Although most participants stated they are interested or potentially interested in participating in a clinical trial to evaluate new treatments for FAs, few had been asked to participate in a trial. Among those who were currently participating in a trial or had considered participation, several barriers to participation emerged, including inconvenience, eligibility criteria, and lack of awareness about clinical trials for FAs. Drug developers, the patient community, and clinicians can work together to improve awareness and accessibility of clinical trials.

### Study strengths and limitations

This study has several key strengths. We interviewed 81 people with a physician-confirmed diagnosis of at least 1 IgE-mediated FA. The interview discussion guides were developed using the National Health Council’s Patient Experience Mapping Toolbox, which was codeveloped by researchers and the patient community. This enabled comprehensive and inclusive input as to IgE-mediated FA patients’ experiences, including (1) FA’s natural history, including signs, symptoms, health system experiences, and diagnosis pathways; (2) disease burden and psychosocial impacts on individuals and families; (3) perspectives on treatment attributes, benefits, and clinical trial participation; and (4) topics and resources that patients and caregivers seek regarding FAs. All study procedures were guided by input from a Steering Committee, which included patient advocates and a clinician.

Despite these strengths, there are limitations when interpreting the study findings. Although we sought to recruit a diverse sample, all interviews and study documents were in English. Thus, the findings may not represent non-English speakers’ experiences living with FAs in the United States. Also, because our sample included predominantly people living in urban and suburban settings, it may not reflect challenges or experiences unique to people living in rural settings.

In addition, although we required physician confirmation of diagnosis, we cannot verify that all are board-certified allergists, and it is possible that certain participants are misdiagnosed with an IgE-mediated FA. Skin prick and blood tests are known to produce false positives. One participant had a diagnosed milk allergy, but our clinician partner suspected they were describing their experience with milk protein proctocolitis. Separately, a patient with multiple FAs described symptoms that may be undiagnosed eczema rather than an IgE-mediated FA. Although food protein–induced enterocolitis syndrome was an exclusion criterion, it is possible that participants had an undiagnosed non–IgE-mediated allergy such as food protein–induced enterocolitis syndrome, especially those reporting gastrointestinal symptoms in isolation.

Finally, although we sought to interview adolescents as 1 of our 3 strata, many of the adolescents’ parents wished to participate and actively contribute. Caregivers’ participation helped fill in details about experiences the adolescents had as young children or toddlers, such as early exposure, symptoms, and diagnosis. However, their presence may have been a barrier to adolescent participants describing the social, financial, or emotional impacts of FA on themselves or other family members.

### Conclusions

This study provides the clinical community with valuable insights into the full spectrum of concerns faced by patients with IgE-mediated FAs, spanning from prediagnosis to daily management and treatment. Enhancing clinicians’ awareness and understanding of their patients’ specific needs can lead to more personalized care, improved patient experiences, and improved outcomes. Providing educational resources, both by disseminating existing resources and by developing new ones, can empower patients and their families to better understand FAs, available treatments, and other management strategies. A care team approach emphasizing clear communication and psychosocial support would also help meet patients’ unmet needs. Furthermore, collaboration among drug developers, the patient community, and clinicians is needed to address barriers to clinical trial participation, including limited awareness and accessibility.

**Clinical implications**

**Understanding the full range of patient experiences can guide clinicians in providing personalized care and optimizing outcomes. Recognizing the specific challenges and burdens associated with IgE-mediated FAs, both within and outside the health care system, is crucial for clinician care teams. This study identified important topics for clinicians to discuss with patients and highlighted resources that, in many cases, already exist but patients are unaware of.**

## Disclosure statement

This research was supported by a sponsorship from Novartis Pharmaceuticals. All conclusions and opinions expressed in this manuscript are solely those of the authors and do not necessarily reflect the official views of Novartis Pharmaceuticals.

Disclosure of potential conflict of interest: E. Oehrlein and J. Vandigo are employees of Applied Patient Experience, LLC (APX), which has contracts with various nonprofit organizations, pharmaceutical companies, and academic institutions. S. C. Schoch, O. A. Escontrías, and R. Rutta are employees of the National Health Council, a membership organization that receives dues and sponsorships from various funders, both members and sponsors. Please see the National Health Council website for lists of members and sponsors (http://www.nationalhealthcouncil.org/). M. Pistiner has served as a consultant for AAFA, kaleo, DBV Technologies, A-immune, Novartis, Bryn, Anjo, and FoodGraph; received funding from AAFA, AAN, kaleo, DBV Technologies, Aquestive, ARS, Kenvue, National Peanut Board, and Egg Nutrition Center; and is cofounder of AllergyHome and Allergy Certified Training. S. Eftekhari and M. Carver are employees of the 10.13039/100014122Asthma and Allergy Foundation of America, a nonprofit patient organization that has received grants for research and unbranded initiatives from Aimmune, DBV Technologies, Genentech, kaleo, Novartis, Pfizer, and Viatris. J. Marvel is an employee of Novartis Services, Inc, and a shareholder of Novartis Pharmaceuticals Corp. C. L. Pashos received financial support from APX in the conduct of this research. J. Voss is an employee and shareholder of Novartis Pharma AG.

Declaration of generative AI and AI-assisted technologies in the writing process: During the preparation of this work, the author(s) used Grammarly to improve grammar. After using this tool/service, the author(s) reviewed and edited the content as needed and took(s) full responsibility for the publication’s content.

## Supplementary data

Supplementary Table E1
