# Supplementary material for: US food allergy patients’ experiences, priorities, and needs: A qualitative study
Source: J Allergy Clin Immunol Glob. 2025 Apr 22;4(3):100482. doi: 10.1016/j.jacig.2025.100482 (PMC12140940; doi:10.1016/j.jacig.2025.100482)
Supplement: Supplementary Table E1 [file mmc1.docx]

### Supplemental Table 1. Signs and symptoms atypical of IgE-mediated food allergies

| Symptom | Illustrative Quote | Frequency  n (%) |
| --- | --- | --- |
| **Isolated Itchiness** | | |
| Potential for eczema  Itching causing bleeding | **Caregiver of an adolescent** (*milk, egg*): “He would scratch so bad that when I’d get him up in the morning, he would have scratched at night, when I didn’t catch him and be bleeding from scratching so hard. And then he ended up getting infections because of the scratching and stuff.” | 2  (2.5%) |
| **Fever, changing temperature, sweating** | | |
|  | **Adult** (*milk*): “So, after about six hours after eating something with something like that in it, then I’ll spike a fever”  **Adolescent** (*peanut, shellfish*): “I would go through being super hot and sweating and then being super cold.” | 6  (7.4%) |
| **Nose Bleeds** | | |
|  | **Adult** (*peanut, tree nut, shellfish, fish*): “I had very frequent nose bleeds” | 3  (3.7%) |
| **Isolated Gastrointestinal Issues** | | |
| Diarrhea | **Adult** (*milk*): If it actually got into my digestive system, then I would have diarrhea or bouts of diarrhea, which I still have today. I'm very careful on the amount of anything with dairy in it that I use because of the gastrointestinal issues that I end up having.  **Adult** (*wheat*): I have great gut problems. It really can mess up my stomach extremely. | 3  (3.7%) |
| Stomachache | **Adult** (*peanut, tree nut*): Sharp pains in my stomach.  **Adult** (*wheat*): Something felt weird in my tummy. | 11  (13.5%) |
